# Supplementary material for: The Cis-Effect Explained Using Next-Generation QTAIM
Source: Molecules. 2022 Sep 18;27(18):6099. doi: 10.3390/molecules27186099 (PMC9506152; doi:10.3390/molecules27186099)
Supplement: Supplementary file 1 [file molecules-27-06099-s001.zip › molecules-1885066-supplementary.pdf]

SUPPLEMENTARY MATERIALS

The *Cis*-Effect Explained Using Next-Generation QTAIM

Yuting Peng [1], Wenjing Yu [1], Xinxin Feng [1], Tianlv Xu [1], Herbert Früchtl [2], Tanja van Mourik [2], Steven R. Kirk [1,*] and Samantha Jenkins [1,*]

[1] Key Laboratory of Chemical Biology and Traditional Chinese Medicine Research and Key Laboratory of Resource National and Local Joint Engineering Laboratory for New Petro-chemical Materials and Fine Utilization of Resources, College of Chemistry and Chemical Engineering, Hunan Normal University, Changsha 410081, China; pyt@hunnu.edu.cn (Y.P.); 202120122404@hunnu.edu.cn (W.Y.); fengxx@hunnu.edu.cn (X.F.); xutl@hunnu.edu.cn (T.X.); steven.kirk@cantab.net (S.R.K.); samanthajsuman@gmail.com (S.J.)

[2] EaStCHEM School of Chemistry, University of St Andrews, North Haugh, St Andrews, Fife KY16 9ST, Scotland, UK; herbert.fruchtl@st-andrews.ac.uk (H.F.); tanja.vanmourik@st-andrews.ac.uk (T.v.M.)

* Correspondence: steven.kirk@cantab.net (S.R.K.); samanthajsuman@gmail.com (S.J.)

1. Supplementary Materials S1. NG-QTAIM and stress tensor theoretical background and procedure to generate the stress tensor trajectories $\mathbb{T}_\sigma(s)$.

2. Supplementary Materials S2. Experimental data, geometry optimized structures for ethene, doubly substituted ethene and diazene.

3. Supplementary Materials S3. Distance measures for ethene, doubly substituted ethene and diazene.

4. Supplementary Materials S4. Scalar measures (relative energy ΔE) for ethene, doubly substituted ethene and diazene.

5. Supplementary Materials S5. The maximum stress tensor projections $\mathbb{T}_\sigma(s)_{max}$ for ethene, doubly substituted ethene and diazene.

6. Supplementary Materials S6. Tables of the complete C1-C2 *BCP* $\mathbb{U}_\sigma$-space distortion sets for ethene and doubly substituted ethene and diazene.

1. Supplementary Materials S1.

I(i) *QTAIM and stress tensor bond critical point (BCP) properties; ellipticity ε:*

The four types of QTAIM critical points are labeled using the notation (*R*, ω) where *R* is the rank of the Hessian matrix, i.e., the number of distinct non-zero eigenvalues and ω is the signature (the algebraic sum of the signs of the eigenvalues); the (3, -3) [nuclear critical point (*NCP*), a local maximum], (3, -1) and (3, 1) [saddle points, referred to as bond critical points (*BCP*) and ring critical points (*RCP*), respectively] and (3, 3) [the cage critical points (*CCP*)]. In the limit that the forces on the nuclei are zero, an atomic interaction line[1] the line passing through a *BCP* and terminating on two nuclear attractors along which the charge density $\rho(\mathbf{r})$ is locally maximal with respect to nearby lines, becomes a bond-path[2]. The full set of critical points with the bond-paths of a molecule or cluster is referred to as the molecular graph.

- Ellipticity $\varepsilon = |\lambda_1|/|\lambda_2| - 1$.

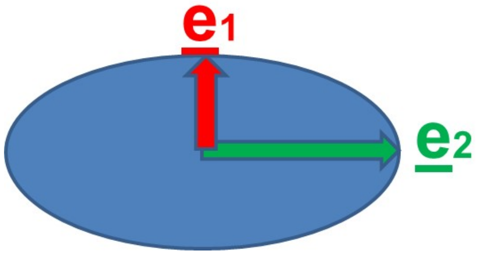

Scheme S1. The cross section through a bond at the bond critical point (*BCP*). The λ_1 and λ_2 eigenvalues with associated eigenvectors $\underline{\mathbf{e}}_1$ and $\underline{\mathbf{e}}_2$ respectively, define the axes of the ellipse and indicate the magnitudes of the least and greatest extents of the distribution of $\rho(\mathbf{r})$.

The ellipticity, ε, defined as $\varepsilon = |\lambda_1|/|\lambda_2| - 1$, quantifies the relative accumulation of the electronic charge density $\rho(\mathbf{r}_b)$ distribution in the two directions $\underline{\mathbf{e}}_1$ and $\underline{\mathbf{e}}_2$ that are perpendicular to the bond-path at a Bond Critical Point (*BCP*) with position $\mathbf{r}_b$. For ellipticity $\varepsilon > 0$, the shortest and longest axes of the elliptical distribution of $\rho(\mathbf{r}_b)$ are associated with the λ_1 and λ_2 eigenvalues, respectively. From the electron-preceding perspective a change in the electronic charge density distribution that defines a chemical bond causes in a change in atomic positions[3]. Bone and Bader later proposed that the direction of motion of the atoms that results from a slightly perturbed structure coincides with the direction of motion of the electrons[4]; this was later confirmed[5,6].

I(ii). *QTAIM bond-path properties; bond-path length (BPL), bond-path curvature, stress tensor eigenvalue $\lambda_{3\sigma}$ and the stress tensor trajectory $T_\sigma(s)$:*

The bond-path length (BPL) is defined as the length of the path traced out by the $\underline{\mathbf{e}}_3$ eigenvector of the Hessian of the total charge density $\rho(\mathbf{r})$, passing through the *BCP*, along which $\rho(\mathbf{r})$ is locally maximal with respect to any neighboring paths. The bond-path curvature separating two bonded nuclei is defined as the dimensionless ratio (BPL - GBL)/GBL, where the BPL is defined to be the bond-path length associated and

GBL is the inter-nuclear separation. The BPL often exceeds the GBL particularly in strained bonding environments[5]. Earlier, one of the current authors hypothesized that a bond-path may possess 1-D, 2-D or a 3-D morphology[7], with 2-D or a 3-D bond-paths associated with a *BCP* with ellipticity $\varepsilon > 0$, being due to the differing degrees of charge density accumulation, of the λ_2 and λ_1 eigenvalues respectively. Bond-paths possessing zero and non-zero values of the bond-path curvature defined by equation **(2)** can be considered to possess 1-D and 2-D topologies respectively. We start by choosing the length traced out in 3-D by the path swept by the tips of the scaled $\underline{\mathbf{e}}_2$ eigenvectors of the λ_2 eigenvalue, the scaling factor being chosen as the ellipticity ε, see **Scheme S1 (a)**.

- Stress tensor eigenvalue $\lambda_{3\sigma}$

This is used as a measure of bond-path instability, for values of $\lambda_{3\sigma} < 0$ and is calculated within the QTAIM partitioning.

The quantum stress tensor $\boldsymbol{\sigma}(\mathbf{r})$ is directly related to the Ehrenfest force by the virial theorem and therefore provides a physical explanation of the low frequency normal modes that accompany structural rearrangements[8]. In this work we use the definition of the stress tensor proposed by Bader to investigate the stress tensor properties within QTAIM[9]. The quantum stress tensor $\boldsymbol{\sigma}(\mathbf{r})$ is used to characterize the mechanics of the forces acting on the electron density distribution in open systems, defined as:

$$\boldsymbol{\sigma}(\mathbf{r}) = -\frac{1}{4}\left[\left(\frac{\partial^2}{\partial \mathbf{r}_i \partial \mathbf{r}'_j} + \frac{\partial^2}{\partial \mathbf{r}'_i \partial \mathbf{r}_j} - \frac{\partial^2}{\partial \mathbf{r}_i \partial \mathbf{r}_j} - \frac{\partial^2}{\partial \mathbf{r}'_i \partial \mathbf{r}'_j}\right) \cdot \gamma(\mathbf{r},\mathbf{r}')\right]_{\mathbf{r}=\mathbf{r}'}, \quad \mathbf{(2)}$$

Where $\gamma(\mathbf{r},\mathbf{r}')$ is the one-body density matrix,

$$\gamma(\mathbf{r},\mathbf{r}') = N\int \Psi(\mathbf{r},\mathbf{r}_2,\ldots,\mathbf{r}_N)\Psi^*(\mathbf{r}',\mathbf{r}_2,\ldots,\mathbf{r}_N)\mathrm{d}\mathbf{r}_2\cdots \mathrm{d}\mathbf{r}_N \quad \mathbf{(3)}$$

The stress tensor is then any quantity $\boldsymbol{\sigma}(\mathbf{r})$, that satisfies equation **(2)** since one can add any divergence-free tensor to the stress tensor without violating this definition.

Earlier, it was found that the stress tensor trajectories $\mathrm{T}_\sigma(s)$ were in line with physical intuition [10].

If we first consider a tiny cube of fluid flowing in 3-D space the stress $\Pi(x, y, z, t)$, a rank-3 tensor field, has nine components [11]: of these the three diagonal components Π_{xx}, Π_{yy}, and Π_{zz} correspond to normal stress. A negative value for these normal components signifies a compression of the cube, conversely a positive value refers to pulling or tension, where more negative/positive values correspond to increased compression/tension of the cube. Diagonalization of the stress tensor $\boldsymbol{\sigma}(\mathbf{r})$, returns the principal electronic stresses Π_{xx}, Π_{yy}, and Π_{zz} that are realized as the stress tensor eigenvalues $\lambda_{1\sigma}$, $\lambda_{2\sigma}$, $\lambda_{3\sigma}$, with corresponding eigenvectors $\underline{\mathbf{e}}_{1\sigma}$, $\underline{\mathbf{e}}_{2\sigma}$, $\underline{\mathbf{e}}_{3\sigma}$ are calculated within the QTAIM partitioning.

Previously, $\lambda_{3\sigma}$ was used to detect the lowering of the symmetry, caused by a torsion about the central C-C

bond in biphenyl, inducing a phase transition [8]. The *BCP*s calculated with QTAIM and stress tensor partitionings will not always coincide, particularly under the application of external force, such as an applied torsion.

I(iii). *The stress tensor trajectory $T_\sigma(s)$ background, interpretation and numerical procedures to generate:*

The changing orientation and characteristics will be undertaken using the stress tensor trajectory space formalism of the bond critical points (*BCP*s). The stress tensor trajectory $T_\sigma(s)$ space U_σ has been previously used to track changing orientation and characteristics of the series of rotational isomers of the S and R stereoisomers of lactic acid [12], the prediction of torquoselectivity in competitive ring-opening reactions [13], elucidating the mechanism of photochromism and fatigue switches [14], the functioning of doped azophenine switches [15] as well as for the reaction pathways of $(H_2O)_5$ [10]. The stress tensor trajectory $T_\sigma(s)$ is intended for use in applications where there will be a finite, i.e. non-zero translation of a given *BCP*, either in real space or the stress tensor trajectory $T_\sigma(s)$ space U_σ. For instance, previously, we examined torsion about the *BCP* located at the fixed 'pivot' of the torsion of the C-C bond linking the two phenyl rings in biphenyl where, as expected, was no significant translation of the central *BCP* [8].
For a given *BCP*, the stress tensor eigenvectors $\{\underline{\mathbf{e}}_{1\sigma}, \underline{\mathbf{e}}_{2\sigma}, \underline{\mathbf{e}}_{3\sigma}\}$ for that *BCP* at the step of the normal mode displacement are used as the projection set for the entire stress tensor trajectory $T_\sigma(s)$. The location in the stress tensor U_σ-space $\mathbf{dr}'(s)$ corresponding to the direction vector $\mathbf{dr}(s)$ in real space is given by $\{(\underline{\mathbf{e}}_{1\sigma}\cdot\mathbf{dr}), (\underline{\mathbf{e}}_{2\sigma}\cdot\mathbf{dr}), (\underline{\mathbf{e}}_{3\sigma}\cdot\mathbf{dr})\}$. The location in the U-space $\mathbf{dr}'(s)$ corresponding to the direction vector $\mathbf{dr}(s)$ in real space is given by $\{(\underline{\mathbf{e}}_{1}\cdot\mathbf{dr}), (\underline{\mathbf{e}}_{2}\cdot\mathbf{dr}), (\underline{\mathbf{e}}_{3}\cdot\mathbf{dr})\}$.

The explanation and numerical procedures for the stress tensor $T_\sigma(s)$ trajectories.

The stress tensor trajectory $T_\sigma(s)$ is constructed exclusively using the frame of reference defined by the eigenvectors $\{\pm\underline{\mathbf{e}}_{1\sigma},\pm\underline{\mathbf{e}}_{2\sigma},\pm\underline{\mathbf{e}}_{3\sigma}\}$ of the stress tensor within the Hessian partitioning, of the total charge density $\rho(\mathbf{r}_b)$ evaluated at the *BCP*, corresponding to the *equilibrium geometry* and is used to construct *all* subsequent points along the $T_\sigma(s)$. This real space frame of reference has been referred to as U_σ-space [12,14–17,10] however, this nomenclature is unnecessarily complex and so we won't use it in the main text. The $T_\sigma(s)$ is constructed using the change in position of the *BCP*, for all displacement steps $\mathbf{dr}$ of the *BCP* in the calculation. Each *BCP* shift vector $\mathbf{dr}$ is mapped to a point $\{(\underline{\mathbf{e}}_{1\sigma}\cdot\mathbf{dr}), (\underline{\mathbf{e}}_{2\sigma}\cdot\mathbf{dr}), (\underline{\mathbf{e}}_{3\sigma}\cdot\mathbf{dr})\}$ in sequence, forming the $T_\sigma(s)$. This mapping is sufficiently symmetry breaking to distinguish stereo-isomers and isotopomers with degenerate relative energies. In contrast, *conventional* QTAIM is confined to the use of scalar measures and therefore can only magnify differences that may exist in relative energies associated with different structures. Conventional QTAIM cannot therefore distinguish stereo-isomers that have degenerate, i.e. equal, relative energies.

Numerical considerations for calculations of the trajectories:

Central to the concept of the trajectories $\mathsf{T}_\sigma(s)$ is the concept of a monotonically increasing sequence parameter s, which may take the form of an increasing integer sequence (0, 1, 2, 3,...) in applications where a set of discrete numbered steps are involved, or a continuous real number. The 3-D stress tensor trajectory $\mathsf{T}_\sigma(s)$ is then defined as an ordered set of points, whose sequence is described by the parameter s. In this application, we used an integer step number for s. We first choose to associate $s = 0$ with a specific reference molecular graph, in this case, the energy minimum structure. For a specific *BCP*, the coordinates associated with each of the points are calculated by evaluating the components of the shift vector $\mathbf{dr} = \mathbf{r_b}(s) - \mathbf{r_b}(s\text{-}1)$ where $\mathbf{r_b}$ indicates the location of the *BCP*, from the previous step to the current step in the reference coordinate frame defined by the eigenvectors $\underline{\mathbf{e}}_{1\sigma}$, $\underline{\mathbf{e}}_{2\sigma}$, $\underline{\mathbf{e}}_{3\sigma}$.
Note: for displaying the QTAIM $\mathsf{T}(s)$ and stress tensor trajectories $\mathsf{T}_\sigma(s)$, large steps that can occur at the beginning or end of a stress tensor trajectory $\mathsf{T}_\sigma(s)$ may swamp the appearance of the stress tensor trajectory $\mathsf{T}_\sigma(s)$. To solve this we temporarily filter these steps before including them back in to correctly calculate the U_σ-space stress tensor trajectory $\mathsf{T}_\sigma(s)$.
The calculation of the $\mathsf{T}_\sigma(s)$ is made easier if the code which produces the list of structures corresponding to points along each step of the torsion (CW) ($-90.0° \leq \theta \leq 0.0°$) and counterclockwise CCW ($0.0° \leq \theta \leq +90.0°$) generates these structures at regularly-spaced points along. The consequence of this desirable characteristic is that there are few or no large changes or 'spikes' in the magnitude of the *BCP* shift vector **dr** i.e. $\Delta\mathbf{dr}$, between path step s and $s + 1$. Such anomalies occur because some path-following algorithms may employ occasional small predictor-corrector steps that are at least an order of magnitude smaller than standard steps. In this analysis it is observed that such intermittent relatively small steps in turn cause very small shifts **dr** to be interspersed between longer runs of larger changes, causing 'spike' noise in the otherwise smooth trajectories $\mathsf{T}(s)$. Such 'spikes', which usually only consist of a single spurious point deviating from the locally smooth stress tensor trajectory, can make potentially large spurious contributions to the stress tensor trajectory $\mathsf{T}_\sigma(s)$ and may be safely filtered. This filtering process was carried out manually in the current work.
In future to avoid the need to manually filter out these 'spikes', a combination of criteria are recommended for automated rejection of inclusion of a specific point into the trajectories $\mathsf{T}_\sigma(s)$:

1. If the magnitude of the **dr** associated with any current stress tensor trajectory point is less than 50% of the average of the corresponding **dr** values associated with the immediately preceding point and the immediately following point, the current point is filtered out as a 'spike'.
2. Abrupt changes in direction in the stress tensor trajectory $\mathsf{T}_\sigma(s)$, e.g. turning by more than 60^0 from one stress tensor trajectory $\mathsf{T}_\sigma(s)$ step to the next cause the current point to be labelled as a 'spike'.

These two rules taken together are referred to as the 'turn' filter. These rules can be repeatedly applied across

multiple ‘passes’ through the stress tensor trajectory data as necessary.
It has been observed that the magnitudes of the steps **dr** naturally tend to slowly decrease toward the end of paths, corresponding to a slowed approach to an end minimum, and the corresponding part of the stress tensor trajectory T_σ (s) turns toward the U_σ-space origin. A combination of the criteria mentioned above may be deployed to retain these parts of the stress tensor trajectory $T_\sigma(s)$.
An alternative Kolmogorov-Zurbenko [18] data smoothing filter may also be applied.

1. Percentage deviation Δ**dr** of the magnitude of the **dr** from a moving average calculated along the stress tensor trajectory $T_\sigma(s)$ exceeding a specific value should be 10% or less.
2. Abrupt changes in direction in the stress tensor trajectory $T_\sigma(s)$, e.g. turning by more than 60° from one stress tensor trajectory $T_\sigma(s)$ step to the next.
It has been observed that the magnitudes of the steps **dr** naturally tend to slowly decrease toward the end of paths, corresponding to a slowed approach to an end minimum, and the corresponding part of the stress tensor trajectory $T_\sigma(s)$ turns toward the U_σ-space origin. A combination of the criteria mentioned above may be deployed to retain these parts of the stress tensor trajectory $T_\sigma(s)$. A range of alternative traditional 'denoising' algorithms may also conceivably be usefully deployed.

I(iv). *Explanation of the stress tensor trajectory $T_\sigma(s)$ in U_σ-space:*

The calculation of the stress tensor trajectory $T_\sigma(s)$ for the torsional *BCP* uses the frame of reference defined by the stress tensor eigenvectors $\{\pm\underline{\mathbf{e}}_{1\sigma},\pm\underline{\mathbf{e}}_{2\sigma},\pm\underline{\mathbf{e}}_{3\sigma}\}$ at the torsional *BCP*. This frame of reference is referred to as the stress tensor trajectory space (U_σ-space) is also used to construct *all* subsequent points along the $T_\sigma(s)$ for dihedral torsion angles in the range -90.0° ≤ θ ≤+90.0°, where θ = 0.0° usually corresponds to the minimum energy geometry. We adopt the convention that CW circular rotations for the range -90.0° ≤ θ ≤ 0.0° and CCW circular rotations for the range 0.0° ≤ θ ≤+90.0°. The change in position of the *BCP* in the U_σ-space is used to construct the $T_\sigma(s)$ using the displacement steps **dr** of the calculation. Each <u>finite</u> *BCP* displacement vector **dr** is mapped to a point $\{(\underline{\mathbf{e}}_{1\sigma}\cdot\mathbf{dr}), (\underline{\mathbf{e}}_{2\sigma}\cdot\mathbf{dr}), (\underline{\mathbf{e}}_{3\sigma}\cdot\mathbf{dr})\}$ in sequence, forming the $T_\sigma(s)$, that is constructed from the vector dot products (the dot product is a projection: a measure of vectors being parallel to each other) of the stress tensor $T_\sigma(s)$ eigenvector components evaluated at the *BCP*. The projections of the *BCP* displacements **dr** are associated with the bond torsion: $\underline{\mathbf{e}}_{1\sigma}.\mathbf{dr}$ → bond-twist, $\underline{\mathbf{e}}_{2\sigma}.\mathbf{dr}$ → bond-flexing and $\underline{\mathbf{e}}_{3\sigma}.\mathbf{dr}$ → bond-axiality [12,14,15,10,16,17,19].
The gap that we observe for the $T_\sigma(s)$ between the CCW and CW torsions at a torsion θ = 0.0° is due to their *BCP* shift vectors **dr** being oppositely directed and of non-zero magnitude. For example for a CCW torsion starting at θ = 0.0° with a finite *BCP* shift **dr = {+0.01, 0.00, 0.00}**, the corresponding CW finite *BCP* shift **dr ≈ {-0.01, 0.00, 0.00},** giving a U_σ-space separation of the $T_\sigma(s)$ of the CCW and CW torsions at a torsion θ = 0.0° of approximately 0.01-(-0.01) = 0.02. This mapping is sufficiently symmetry breaking to enable the $T_\sigma(s)$

of S and R stereoisomers at the equilibrium configuration to be distinguished, in contrast to conventional *scalar* QTAIM. The $\mathsf{T}_{\sigma}(s)$ comprises a series of contiguous points as a 3-D vector path displaying the effect of the structural change and is analyzed here in terms of the CW and CCW directions of bond torsion. The $\underline{\mathbf{e}}_{1\sigma}$ corresponds to the *most* preferred direction of charge density $\rho(\mathbf{r})$ accumulation and therefore the most facile direction in the plane perpendicular to the bond-path, where bond-torsion about the *BCP* does not involve structural distortion in the form of any increase in bond-path length from the straight-line bonded separation. A value of $\{\underline{\mathbf{e}}_{1\sigma}\cdot\mathbf{dr}\}_{\mathbf{max}} = 0.0$ corresponds to a constant orientation of the $\underline{\mathbf{e}}_{1\sigma}$ eigenvector in real space and therefore constant bond-path torsion (bond-twist). The subscript "**max**" corresponds to the difference between the minimum and maximum value of the projection of the *BCP* shift **dr** onto $\underline{\mathbf{e}}_{1\sigma}$ or $\underline{\mathbf{e}}_{2\sigma}$ or $\underline{\mathbf{e}}_{3\sigma}$ along the entire stress tensor trajectory $\mathsf{T}_{\sigma}(s)$, see **Tables 1-5**. We will denote the maximum stress tensor projections $\mathsf{T}_{\sigma}(s)_{max}$ = {bond-twist$_{max}$, bond-flexing$_{max}$, bond-anharmonicity$_{max}$}; these quantities therefore define the dimensions of a 'bounding box' around each $\mathsf{T}_{\sigma}(s)$. The $\underline{\mathbf{e}}_{2\sigma}$ corresponds to the *least* preferred, i.e. the least readily distorted, direction of charge density $\rho(\mathbf{r})$ accumulation and therefore least facile direction in the plane perpendicular to the bond-path. This is because bond-flexing requires a greater structural distortion than bond-twist (torsion) in the form of an increase in bond-path length from the straight-line bonded separation. A value $\{\underline{\mathbf{e}}_{2\sigma}\cdot\mathbf{dr}\}_{\mathbf{max}} = 0.0$ corresponds to constant bond-flexing in real space. A value of $\{\underline{\mathbf{e}}_{3\sigma}\cdot\mathbf{dr}\}_{\mathbf{max}} > 0.0$ indicates the bond-axiality corresponding to a changing *BCP* shift **dr** in real space relative to the bond-path, and therefore greater freedom for the *BCP* to slide along the bond-path; note the bond-axiality axis label in **Figures 2**. Conversely, $\{\underline{\mathbf{e}}_{3\sigma}\cdot\mathbf{dr}\}_{\mathbf{max}} = 0.0$ corresponds to a constant *BCP* shift **dr** in real space along the bond-path and therefore an absence of bond-axiality.

2. Supplementary Materials S2. Experimental data, geometry optimized structures.

Table S2. Experimental data for the energy differences between the *cis*- and *trans*-isomers in kcal mol^{-1}. No data could be found for the Cl_2-diazene species. Note that the energy difference for H_2-diazene is quoted as a negative number because the *trans*-isomer is more energetically stable than the *cis*-isomer.

Species	*Compound (gas)*	*Energy difference (kcal mol^{-1})*
F_2-ethene	HFC=CFH	1.080 ± 0.120
Cl_2-ethene	HClC=CClH	0.650 ± 0.070
H_2-diazene	HN=NH	-3.050 ± 0.400
F_2-diazene	FN=NF	3.000 ± 0.300
Cl_2-diazene	ClN=NCl	---

Optimized geometries of the considered molecules (XYZ format: coordinate units are Ångstrom)

```
6
Ethene geom opt
C   0.0000000000    0.0000000000   0.0000000000
C   1.3335168576    0.0000000000   0.0000000000
H  -0.5630666548    0.9146367975   0.0000000000
H  -0.5630666548   -0.9146367975   0.0000000000
H   1.8965835124   -0.9146367975   0.0000000000
H   1.8965835124    0.9146367975   0.0000000000

6
Cis-difluoroethene geom opt
C   0.0000000000    0.0000000000   0.0000000000
C   1.3235138900   -0.0000000000  -0.0000000000
F  -0.7049023468    1.1131702584   0.0000000000
H  -0.5783578339   -0.8986858780  -0.0000000337
H   1.9018717242   -0.8986858778  -0.0000000238
F   2.0284162368    1.1131702586  -0.0000000179

6
Trans-difluoroethene geom opt
C   0.0000000000    0.0000000000   0.0000000000
C   1.3231024961   -0.0000000000   0.0000000000
H  -0.6166972691    0.8734323481   0.0000000000
F  -0.6581584863   -1.1471189104   0.0000000045
H   1.9397997622   -0.8734323503  -0.0000000004
F   1.9812609847    1.1471189081  -0.0000000054

6
Cis-dichloroethene geom opt
C   0.0000000000    0.0000000000   0.0000000000
C   1.3275840786    0.0000000000   0.0000000000
Cl -0.9938039492    1.4065854239   0.0000000000
H  -0.5415863730   -0.9217832279  -0.0000000069
H   1.8691704516   -0.9217832279   0.0000000041
Cl  2.3213880278    1.4065854239  -0.0000000268

6
Trans-dichloroethene geom opt
C   0.0000000000    0.0000000000   0.0000000000
C   1.3255411061   -0.0000000000   0.0000000000
H  -0.5985438068    0.8846416889  -0.0000000000
Cl -0.8955809518   -1.4816308817   0.0000000051
H   1.9240849121   -0.8846416894   0.0000000014
Cl  2.2211220584    1.4816308810  -0.0000000022

4
Cis-N2H2 geom opt
N   0.0000000000    0.0000000000   0.0000000000
N   1.2367424133   -0.0000000000  -0.0000000000
H  -0.3878128704    0.9374428179  -0.0000000000
```

```
H   1.6245574260    0.9374419856  -0.0000004796

4
Trans-N2H2 geom opt
N   0.0000000000    0.0000000000   0.0000000000
N   1.2360751764   -0.0000000000   0.0000000000
H  -0.3037408056    0.9640380580   0.0000000000
H   1.5398159305   -0.9640380739  -0.0000001484

4
Cis-N2F2 geom opt
N   0.0000000000    0.0000000000   0.0000000000
N   1.2157704749    0.0000000000   0.0000000000
F  -0.5401037735    1.2120056725   0.0000000000
F   1.7558743404    1.2120061360   0.0000000000

4
Trans-N2F2 geom opt
N   0.0000000000    0.0000000000   0.0000000000
N   1.2152349472    0.0000000000   0.0000000000
F  -0.3766507440    1.2740159183   0.0000000000
F   1.5918857986   -1.2740158930   0.0000000000

4
Cis-N2Cl2 geom opt
N   0.0000000000    0.0000000000   0.0000000000
N   1.2070260416    0.0000000000   0.0000000000
Cl -0.9015654100    1.4751311507   0.0000000000
Cl  2.1085889132    1.4751333847   0.0000000000

4
Trans-N2Cl2 geom opt
N   0.0000000000    0.0000000000   0.0000000000
N   1.2118269343    0.0000000000   0.0000000000
Cl -0.5911910867    1.6236941240   0.0000000000
Cl  1.8030180871   -1.6236941048  -0.0000016632
```

3. Supplementary Materials S3. Distance measures for substituted ethene and diazene.

Table S3(a). The distance measures of the unsubstituted and the doubly substituted ethene for values of the torsion θ = 0.0º. Values of the inter-nuclear separations are referred to as the geometric bond-lengths (GBL) and bond-path lengths (BPL) (in a.u.).

ethene

	GBL	BPL		
			(C1-*BCP*,*BCP*-C2)	
C1-C2 *BCP*	2.5200	2.5200	(1.2600, 1.2600)	
			(C1-*BCP*,*BCP*-H3)	(C1-*BCP*,*BCP*-H4)
C1-H3 *BCP*	2.0297	2.0088	(1.2526, 0.7562)	(---,---)
C1-H4 *BCP*	2.0297	2.0088	(---,---)	(1.2526, 0.7562)
			(C2-*BCP*,*BCP*-H5)	(C2-*BCP*,*BCP*-H6)
C2-H5 *BCP*	2.0297	2.0088	(1.2526, 0.7562)	(---,---)
C2-H6 *BCP*	2.0297	2.0088	(---,---)	(1.2526, 0.7562)

cis-F_2-ethene

			(C1-*BCP*,*BCP*-C2)	
C1-C2 *BCP*	2.5011	2.5050	(1.2525, 1.2525)	
			(C1-*BCP*,*BCP*-F3)	(C1-*BCP*,*BCP*-H4)
C1-F3 *BCP*	2.4899	2.4899	(0.7856, 1.7043)	(---,---)
C1-H4 *BCP*	2.0196	1.9981	(---,---)	(1.2873, 0.7108)
			(C2-*BCP*,*BCP*-H5)	(C2-*BCP*,*BCP*-F6)
C2-H5 *BCP*	2.0196	1.9981	(1.2873, 0.7108)	(---,---)
C2-F6 *BCP*	2.4899	2.4899	(---,---)	(0.7856, 1.7043)

trans-F_2-ethene

			(C1-*BCP*,*BCP*-C2)	
C1-C2 *BCP*	2.5003	2.5018	(1.2509, 1.2509)	
			(C1-*BCP*,*BCP*-H3)	(C1-*BCP*,*BCP*-F4)
C1-H3 *BCP*	2.0205	1.9990	(1.2926, 0.7064)	(---,---)
C1-F4 *BCP*	2.4992	2.4992	(---,---)	(0.7880, 1.7112)
			(C2-*BCP*,*BCP*-H5)	(C2-*BCP*,*BCP*-F6)
C2-H5 *BCP*	2.0205	1.9990	(1.2926, 0.7064)	(---,---)
C2-F6 *BCP*	2.4992	2.4992	(---,---)	(0.7880, 1.7112)

cis-Cl_2-ethene

			(C1-*BCP*,*BCP*-C2)	
C1-C2 *BCP*	2.5088	2.5122	(1.2561, 1.2561)	
			(C1-*BCP*,*BCP*-Cl3)	(C1-*BCP*,*BCP*-H4)
C1-Cl3 *BCP*	3.2546	3.2548	(1.3321, 1.9227)	(---,---)
C1-H4 *BCP*	2.0203	1.9990	(---,---)	(1.2875, 0.7115)
			(C2-*BCP*,*BCP*-H5)	(C2-*BCP*,*BCP*-Cl6)
C2-H5 *BCP*	2.0203	1.9990	(1.2875, 0.7115)	(---,---)
C2-Cl6 *BCP*	3.2546	3.2548	(---,---)	(1.3321, 1.9227)

trans-Cl_2-ethene

			(C1-*BCP*,*BCP*-C2)	
C1-C2 *BCP*	2.5049	2.5066	(1.2533, 1.2533)	
			(C1-*BCP*,*BCP*-H3)	(C1-*BCP*,*BCP*-Cl4)
C1-H3 *BCP*	2.0184	1.9970	(1.2933, 0.7037)	(---,---)
C1-Cl4 *BCP*	3.2716	3.2717	(---,---)	(1.3333, 1.9384)
			(C2-*BCP*,*BCP*-H5)	(C2-*BCP*,*BCP*-Cl6)
C2-H5 *BCP*	2.0184	1.9970	(1.2933, 0.7037)	(---,---)
C2-Cl6 *BCP*	3.2716	3.2717	(---,---)	(1.3333, 1.9384)

Table S3(b). The distance measures of the doubly substituted diazene for values of the torsion θ = 0.0º. Values of the inter-nuclear separations are referred to as the geometric bond-lengths (GBL) and bond-path lengths (BPL) (in a.u.).

cis-diazene

Isomer	GBL	BPL	(N1-*BCP*,*BCP*-N2)	(N1-*BCP*,*BCP*-H3)	(N2-*BCP*,*BCP*-H4)
H₂					
N1-N2 *BCP*	2.3371	2.3404	(1.1702, 1.1702)	(---,---)	(---,---)
N1-H3 *BCP*	1.9171	1.8921	(---,---)	(1.4264, 0.4657)	(---,---)
N2-H4 *BCP*	1.9171	1.8921	(---,---)	(---,---)	(1.4264, 0.4657)
F₂				(N1-*BCP*,*BCP*-F3)	(N2-*BCP*,*BCP*-F4)
N1-N2 *BCP*	2.2975	2.3118	(1.1559, 1.1559)	(---,---)	(---,---)
N1-F3 *BCP*	2.5075	2.5078	(---,---)	(1.0410, 1.4668)	(---,---)
N2-F4 *BCP*	2.5075	2.5078	(---,---)	(---,---)	(1.0410, 1.4668)
Cl₂				(N1-*BCP*,*BCP*-Cl3)	(N2-*BCP*,*BCP*-Cl4)
N1-N2 *BCP*	2.2809	2.2932	(1.1466, 1.1466)	(---,---)	(---,---)
N1-Cl3 *BCP*	3.2670	3.2715	(---,---)	(1.6055, 1.6660)	(---,---)
N2-Cl4 *BCP*	3.2670	3.2715	(---,---)	(---,---)	(1.6055, 1.6660)

trans-diazene

Isomer	GBL	BPL	(N1-*BCP*,*BCP*-N2)	(N1-*BCP*,*BCP*-H3)	(N2-*BCP*,*BCP*-H4)
H₂					
N1-N2 *BCP*	2.3358	2.3370	(1.1685, 1.1685)	(---,---)	(---,---)
N1-H3 *BCP*	1.9101	1.8839	(---,---)	(1.4296, 0.4543)	(---,---)
N2-H4 *BCP*	1.9101	1.8839	(---,---)	(---,---)	(1.4296, 0.4543)
F₂				(N1-*BCP*,*BCP*-F3)	(N2-*BCP*,*BCP*-F4)
N1-N2 *BCP*	2.2965	2.2994	(1.1497, 1.1497)	(---,---)	(---,---)
N1-F3 *BCP*	2.5106	2.5110	(---,---)	(1.0402, 1.4708)	(---,---)
N2-F4 *BCP*	2.5106	2.5110	(---,---)	(---,---)	(1.0402, 1.4708)
Cl₂				(N1-*BCP*,*BCP*-Cl3)	(N2-*BCP*,*BCP*-Cl4)
N1-N2 *BCP*	2.2900	2.2932	(1.1466, 1.1466)	(---,---)	(---,---)
N1-Cl3 *BCP*	3.2654	3.2678	(---,---)	(1.6042, 1.6636)	(---,---)
N2-Cl4 *BCP*	3.2654	3.2678	(---,---)	(---,---)	(1.6042, 1.6636)

4. Supplementary Materials S4. Scalar measures (relative energy ΔE) for substituted ethene and diazene

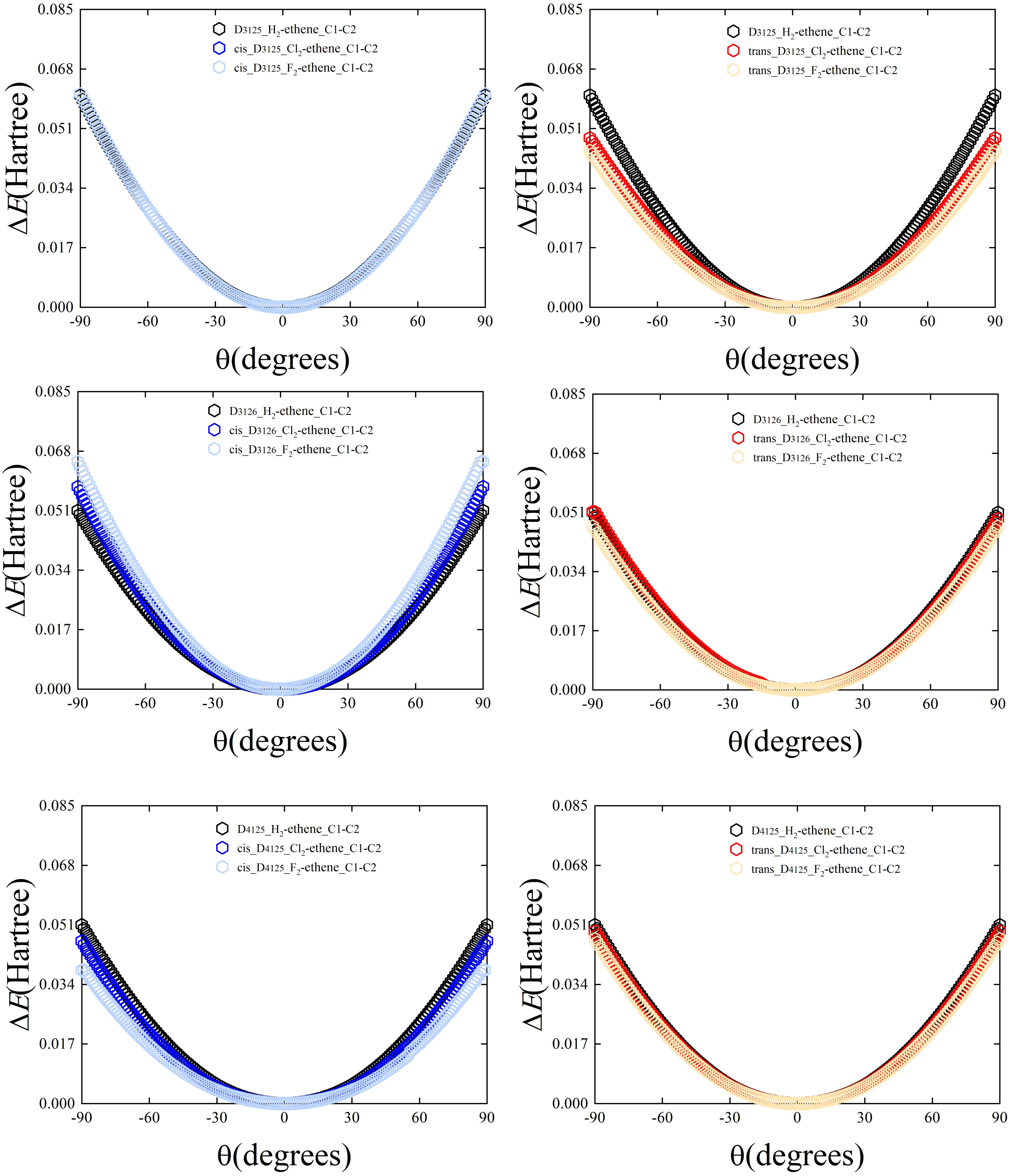

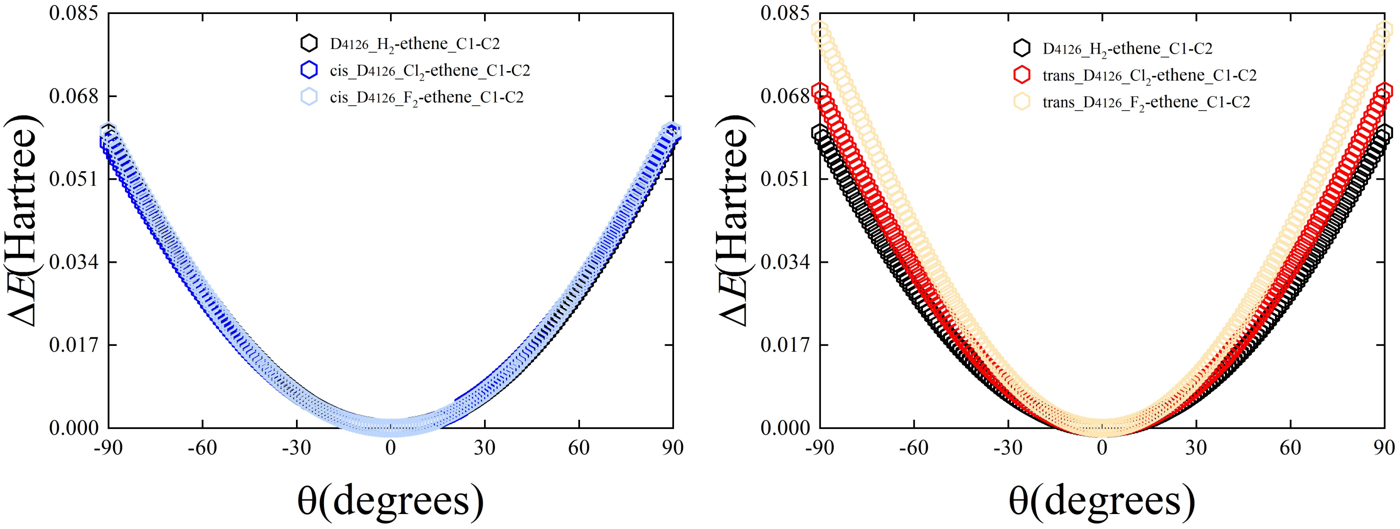

Figure S4(a). The variation of the relative energy ΔE for the clockwise (CW) (-90.0º ≤ θ ≤ 0.0º) and counter-clockwise (CCW) (0.0º ≤ θ ≤ +90.0º) torsion θ isomers of the C1-C2 *BCP* of the unsubstituted and the doubly substituted ethene for the U_σ-space chirality using the D3125 isomer, D3126 isomer, D4125 isomer and D4126 isomer. The unsubstituted-isomers for H_2-ethene (black), the cis-isomers for Cl_2-ethene (dark-blue), F_2-ethene (light-blue) and the *trans*-isomers for Cl_2-ethene (red), F_2-ethene (light-red) substituents are presented.

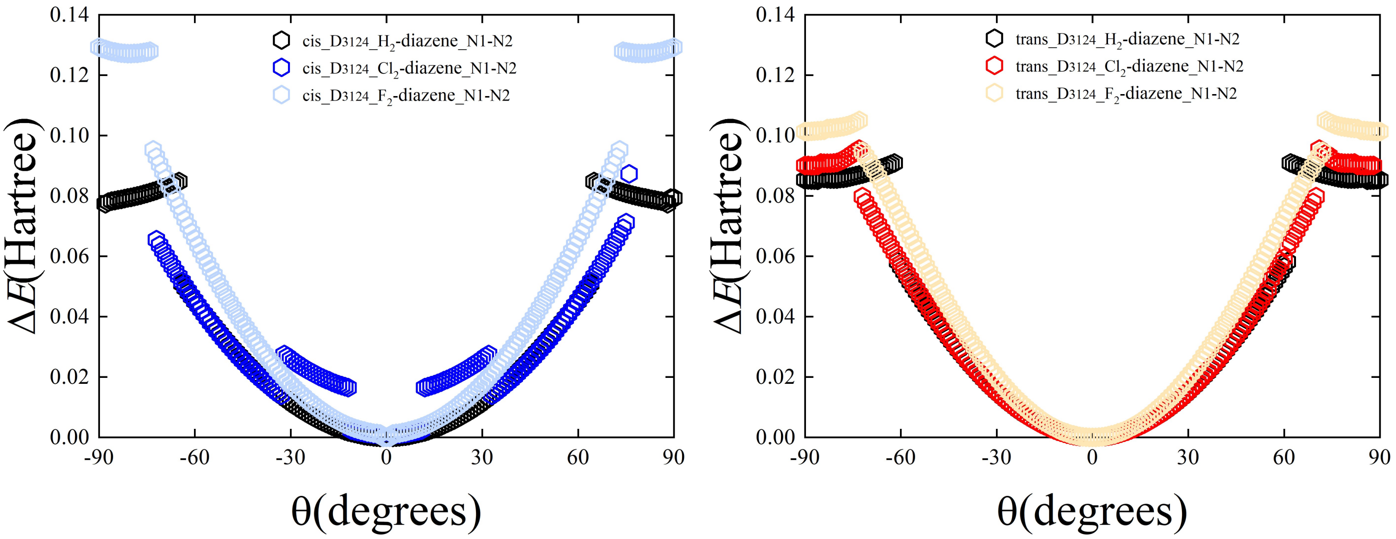

Figure S4(b). The variation of the relative energy ΔE for the CW and CCW torsion θ isomers of the N1-N2 *BCP* of the doubly substituted diazene for the U_σ-space chirality using D3124 isomer. The cis-isomers for H_2-diazene (black) Cl_2-diazene (dark-blue), F_2-diazene (light-blue) and the *trans*-isomers for H_2-diazene (black), Cl_2-diazene (red), F_2-diazene (light-red) substituents are presented.

5. Supplementary Materials S5. The maximum stress tensor projections $\mathrm{T}_{\sigma}(s)_{\max}$ for substituted ethene and diazene

Table S5(a). The maximum stress tensor projections $\mathrm{T}_{\sigma}(s)_{\max}$ = {bond-twist$_{\max}$, bond-flexing$_{\max}$, bond-axiality$_{\max}$} of the torsion C1-C2 *BCP* of the unsubstituted and the doubly substituted ethene; all entries have been multiplied by 10^3.

	{bond-twist$_{\max}$, bond-flexing$_{\max}$, bond-axiality$_{\max}$}	
Isomer	**CW**	**CCW**
	H_2	
D_{3125}	{0.421621, 1.749653, 0.538535}	{0.420658, 1.749885, 0.537814}
D_{3126}	{1.556154, 1.798145, 0.587711}	{1.556184, 1.798204, 0.587890}
D_{4125}	{1.582568, 1.807992, 0.595062}	{1.588166, 1.808127, 0.595067}
D_{4126}	{0.421531, 1.749428, 0.539150}	{0.421699, 1.749310, 0.538772}

	cis-ethene		*trans-ethene*	
	**CW**	**CCW**	**CW**	**CCW**
		F_2		
D_{3125}	{0.894386, 2.478327, 0.766003}	{0.894951, 2.476430, 0.765981}	{0.633980, 1.614118, 0.490659}	{0.616676, 1.614120, 0.490754}
D_{3126}	{1.811066, 1.843635, 1.114622}	{1.797679, 1.843670, 1.113804}	{1.643756, 1.552269, 1.323383}	{1.644868, 1.552524, 1.323285}
D_{4125}	{1.379801, 1.681487, 0.853123}	{1.379820, 1.680001, 0.852128}	{1.346172, 2.036081, 0.453818}	{1.345872, 2.036362, 0.453826}
D_{4126}	{1.794181, 0.948874, 1.629433}	{1.795607, 0.947292, 1.628733}	{0.969545, 1.521856, 2.211110}	{0.968941, 1.521748, 2.211832}
		Cl_2		
D_{3125}	{0.730759, 2.389589, 0.394047}	{0.871561, 3.342181, 0.938698}	{0.269221, 1.876361, 0.289572}	{0.269308, 1.876577, 0.289551}
D_{3126}	{1.631687, 1.754600, 0.799802}	{1.630753, 1.755480, 0.799574}	{1.764909, 0.939509, 0.948342}	{1.360163, 1.133710, 1.085429}
D_{4125}	{1.598443, 1.390790, 0.802927}	{1.597514, 1.390777, 0.802122}	{1.896013, 2.115174, 0.184863}	{1.896178, 2.114261, 0.184909}
D_{4126}	{0.854010, 1.666286, 0.802408}	{0.466735, 1.778609, 0.336531}	{0.397264, 1.967998, 1.206856}	{0.397590, 1.968005, 1.206663}

Table S5(b). The maximum stress tensor projections $\mathrm{T}_{\sigma}(s)_{\max}$ = {bond-twist$_{\max}$, bond-flexing$_{\max}$, bond-axiality$_{\max}$} of the torsion N1-N2 *BCP* of the doubly substituted diazene for the D_{3124} isomer; all entries have been multiplied by 10^3.

{bond-twist$_{\max}$, bond-flexing$_{\max}$, bond-axiality$_{\max}$}			
cis-diazene		*trans-diazene*	
Isomer **CW**	**CCW**	**CW**	**CCW**
	H_2		
{4.801404, 2.787503, 5.635097}	{4.809519, 2.787666, 5.641851}	{4.856140, 3.448154, 5.073475}	{4.854382, 3.448856, 5.072910}
	F_2		
{3.172154, 3.219492, 0.505581}	{3.161382, 3.210634, 0.508708}	{4.583991, 4.100396, 5.827743}	{4.584225, 4.100534, 5.827903}
	Cl_2		
{1.475864, 1.395215, 0.849856}	{1.476064, 1.401934, 0.850026}	{25.904873, 10.353290, 32.153236}	{25.904653, 10.349264, 31.819244}

6. Supplementary Materials S6. Tables of the complete U_σ-space distortion sets for ethene and doubly substituted ethene and diazene.

Table S6(a). The ethene and doubly substituted ethene torsion C1-C2 *BCP* U_σ-space distortion sets {chirality C_σ, bond-flexing F_σ, bond-axiality A_σ}, sum of the four isomers: $\sum\{C_\sigma, F_\sigma, A_\sigma\}$, chirality-helicity function $C_{helicity} = C_\sigma|A_\sigma|$ and the sum of $C_{helicity}$ over the four isomers $\sum C_{helicity}$ for each of the *cis*- and *trans*-isomers. The four-digit sequence (left column) refers to the atom numbering sequence for the dihedral angles used to construct the $T_\sigma(s)$, see **Scheme 1** of the main text. The values of the total sums in the table correspond to all four isomers, the complete table for all four isomers (D_{3125}, D_{3126}, D_{4125}, D_{4126}), is provided. Values of the $C_{helicity}$ with a magnitude of less than 10^{-5} are considered insignificant. $\{C_\sigma, F_\sigma, A_\sigma\}$

H_2

	$\{C_\sigma, F_\sigma, A_\sigma\}$	$\sum C_{helicity}$
D_{3125}	{-0.00096$[\mathbf{R}_\sigma]$, 0.00023$[\mathbf{S}_\sigma]$, -0.00072$[\mathbf{R}_\sigma]$}	
D_{3126}	{0.00003$[\mathbf{S}_\sigma]$, 0.00006$[\mathbf{S}_\sigma]$, 0.00018$[\mathbf{S}_\sigma]$}	
D_{4125}	{0.00560$[\mathbf{S}_\sigma]$, 0.00013$[\mathbf{S}_\sigma]$, 0.00001$[\mathbf{S}_\sigma]$}	
D_{4126}	{0.00017$[\mathbf{S}_\sigma]$, -0.00012$[\mathbf{R}_\sigma]$, -0.00038$[\mathbf{R}_\sigma]$	
$\sum\{C_\sigma, F_\sigma, A_\sigma\}$	{0.00483$[\mathbf{S}_\sigma]$, 0.00030$[\mathbf{S}_\sigma]$, -0.00091$[\mathbf{R}_\sigma]$}	0 (4.4×10^{-6})$[\mathbf{Q}_\sigma]$

F_2

	cis-ethene $\{C_\sigma, F_\sigma, A_\sigma\}$	$\sum C_{helicity}$	*trans-ethene* $\{C_\sigma, F_\sigma, A_\sigma\}$	$\sum C_{helicity}$
D_{3125}	{0.00056$[\mathbf{S}_\sigma]$, -0.00190$[\mathbf{R}_\sigma]$, -0.00002$[\mathbf{R}_\sigma]$}		{-0.01731$[\mathbf{R}_\sigma]$, 0.00000$[\mathbf{Q}_\sigma]$, 0.00010$[\mathbf{S}_\sigma]$}	
D_{3126}	{-0.01339$[\mathbf{R}_\sigma]$, 0.00004$[\mathbf{S}_\sigma]$, -0.00082$[\mathbf{R}_\sigma]$}		{0.00111$[\mathbf{S}_\sigma]$, 0.00026$[\mathbf{S}_\sigma]$, -0.00010$[\mathbf{R}_\sigma]$}	
D_{4125}	{0.00002$[\mathbf{S}_\sigma]$, -0.00149$[\mathbf{R}_\sigma]$, -0.00100$[\mathbf{R}_\sigma]$}		{-0.00030$[\mathbf{R}_\sigma]$, 0.00028$[\mathbf{S}_\sigma]$, 0.00001$[\mathbf{S}_\sigma]$}	
D_{4126}	{0.00143$[\mathbf{S}_\sigma]$, -0.00158$[\mathbf{R}_\sigma]$, -0.00070$[\mathbf{R}_\sigma]$}		{-0.00060$[\mathbf{R}_\sigma]$, -0.00011$[\mathbf{R}_\sigma]$, 0.00072$[\mathbf{S}_\sigma]$}	
$\sum\{C_\sigma, F_\sigma, A_\sigma\}$	{-0.01138$[\mathbf{R}_\sigma]$, -0.00493$[\mathbf{R}_\sigma]$, -0.00253$[\mathbf{R}_\sigma]$}	-0.00003$[\mathbf{R}_\sigma,\mathbf{R}_\sigma]$	{-0.01710$[\mathbf{R}_\sigma]$, 0.00043$[\mathbf{S}_\sigma]$, 0.00073$[\mathbf{S}_\sigma]$}	-0.00001$[\mathbf{R}_\sigma,\mathbf{S}_\sigma]$

Cl_2

	cis-ethene $\{C_\sigma, F_\sigma, A_\sigma\}$	$\sum C_{helicity}$	*trans-ethene* $\{C_\sigma, F_\sigma, A_\sigma\}$	$\sum C_{helicity}$
D_{3125}	{0.14080$[\mathbf{S}_\sigma]$, 0.95259$[\mathbf{S}_\sigma]$, 0.54465$[\mathbf{S}_\sigma]$}		{0.00009$[\mathbf{S}_\sigma]$, 0.00022$[\mathbf{S}_\sigma]$, -0.00002$[\mathbf{R}_\sigma]$}	
D_{3126}	{-0.00093$[\mathbf{R}_\sigma]$, 0.00088$[\mathbf{S}_\sigma]$, -0.00023$[\mathbf{R}_\sigma]$}		{-0.40475$[\mathbf{R}_\sigma]$, 0.19420$[\mathbf{S}_\sigma]$, 0.13709$[\mathbf{S}_\sigma]$}	
D_{4125}	{-0.00093$[\mathbf{R}_\sigma]$, -0.00001$[\mathbf{R}_\sigma]$, -0.00081$[\mathbf{R}_\sigma]$}		{0.00017$[\mathbf{S}_\sigma]$, -0.00091$[\mathbf{R}_\sigma]$, 0.00005$[\mathbf{S}_\sigma]$}	
D_{4126}	{-0.38727$[\mathbf{R}_\sigma]$, 0.11232$[\mathbf{S}_\sigma]$, -0.46588$[\mathbf{R}_\sigma]$}		{0.00033$[\mathbf{S}_\sigma]$, 0.00001$[\mathbf{S}_\sigma]$, -0.00019$[\mathbf{R}_\sigma]$}	
$\sum\{C_\sigma, F_\sigma, A_\sigma\}$	{-0.24833$[\mathbf{R}_\sigma]$, 1.06578$[\mathbf{S}_\sigma]$, 0.07774$[\mathbf{S}_\sigma]$}	-0.0193$[\mathbf{R}_\sigma,\mathbf{S}_\sigma]$	{-0.40417$[\mathbf{R}_\sigma]$, 0.19351$[\mathbf{S}_\sigma]$, 0.13692$[\mathbf{S}_\sigma]$}	-0.0553$[\mathbf{R}_\sigma,\mathbf{S}_\sigma]$

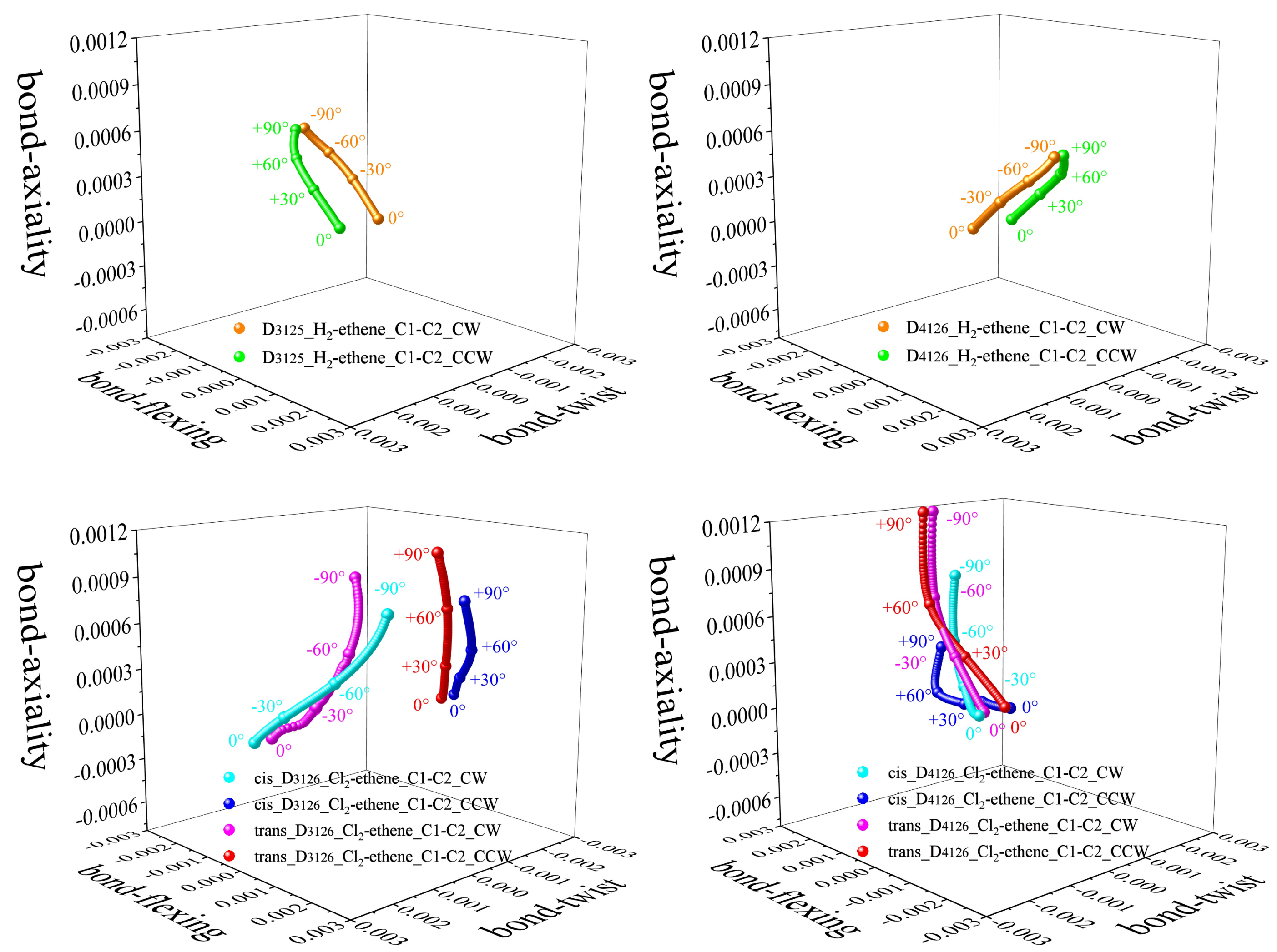

Figure S6(a). The unsubstituted and the doubly substituted ethene C1-C2 *BCP* stress tensor trajectories $\mathsf{T}_{\sigma}(s)$ for the Cartesian CW and CCW torsions for *cis*- and *trans*-isomers of U_{σ}-space isomers the H_2-ethene and Cl_2-ethene**.**

Table S6(b). The doubly substituted diazene torsion C1-C2 *BCP* $\mathbb{U}_\sigma$-space distortion sets {$\mathbb{C}_\sigma$, $\mathbb{F}_\sigma$, $\mathbb{A}_\sigma$}. Note that *cis*- and *trans*- correspond to the geometric isomers, see **Scheme 1**. The complete table for the D_{3124} isomer, is provided in the following table. Values of the $\mathbb{C}_{helicity}$ with a magnitude of less than 10^{-5} are considered insignificant.

cis-diazene {$\mathbb{C}_\sigma$, $\mathbb{F}_\sigma$, $\mathbb{A}_\sigma$}	$\mathbb{C}_{helicity}$	*trans-diazene* {$\mathbb{C}_\sigma$, $\mathbb{F}_\sigma$, $\mathbb{A}_\sigma$}	$\mathbb{C}_{helicity}$
		H₂	
{0.00812[**S**$_\sigma$], 0.00016[**S**$_\sigma$], 0.00675[**S**$_\sigma$]}	0.0001[**S**$_\sigma$,**S**$_\sigma$]	{-0.00176[**R**$_\sigma$], 0.00070[**S**$_\sigma$], -0.00057[**R**$_\sigma$]}	0 (-9.94×10^{-7})
		F₂	
{-0.01077[**R**$_\sigma$], -0.00886[**R**$_\sigma$], 0.00313[**S**$_\sigma$]}	-0.00003[**R**$_\sigma$,**S**$_\sigma$]	{0.00023[**S**$_\sigma$], 0.00014[**S**$_\sigma$], 0.00016[**S**$_\sigma$]}	0 (3.74×10^{-8})
		Cl₂	
{0.00020[**S**$_\sigma$], 0.00672[**S**$_\sigma$], 0.00017[**S**$_\sigma$]}	0 (3.39×10^{-8})	{-0.00022[**R**$_\sigma$], -0.00402[**R**$_\sigma$], -0.33399[**R**$_\sigma$]}	-0.0001[**R**$_\sigma$,**R**$_\sigma$]

References

1. Bader, R.F.W. A Bond Path: A Universal Indicator of Bonded Interactions. *J. Phys. Chem. A* **1998**, *102*, 7314–7323, doi:10.1021/jp981794v.
2. Bader, R.F.W. Bond Paths Are Not Chemical Bonds. *J. Phys. Chem. A* **2009**, *113*, 10391–10396, doi:10.1021/jp906341r.
3. Nakatsuji, H. Common Nature of the Electron Cloud of a System Undergoing Change in Nuclear Configuration. *J. Am. Chem. Soc.* **1974**, *96*, 24–30, doi:10.1021/ja00808a004.
4. Bone, R.G.A.; Bader, R.F.W. Identifying and Analyzing Intermolecular Bonding Interactions in van Der Waals Molecules. *J. Phys. Chem.* **1996**, *100*, 10892–10911.
5. Jenkins, S.; Heggie, M.I. Quantitative Analysis of Bonding in 90° Partial Dislocation in Diamond. *J. Phys. Condens. Matter* **2000**, *12*, 10325–10333, doi:10.1088/0953-8984/12/49/3.
6. Ayers, P.W.; Jenkins, S. An Electron-Preceding Perspective on the Deformation of Materials. *J. Chem. Phys.* **2009**, *130*, 154104, doi:10.1063/1.3098140.
7. Jenkins, S.; Morrison, I. The Chemical Character of the Intermolecular Bonds of Seven Phases of Ice as Revealed by Ab Initio Calculation of Electron Densities. *Chem. Phys. Lett.* **2000**, *317*, 97–102.
8. Jenkins, S.; Maza, J.R.; Xu, T.; Jiajun, D.; Kirk, S.R. Biphenyl: A Stress Tensor and Vector-Based Perspective Explored within the Quantum Theory of Atoms in Molecules. *Int. J. Quantum Chem.* **2015**, *115*, 1678–1690, doi:10.1002/qua.25006.
9. Bader, R.F.W. Quantum Topology of Molecular Charge Distributions. III. The Mechanics of an Atom in a Molecule. *J. Chem. Phys.* **1980**, *73*, 2871–2883, doi:10.1063/1.440457.
10. Xu, T.; Farrell, J.; Momen, R.; Azizi, A.; Kirk, S.R.; Jenkins, S.; Wales, D.J. A Stress Tensor Eigenvector Projection Space for the (H2O)5 Potential Energy Surface. *Chem. Phys. Lett.* **2017**, *667*, 25–31, doi:10.1016/j.cplett.2016.11.028.
11. Wyatt, R.E. *Quantum Dynamics with Trajectories*; Interdisciplinary Applied Mathematics; 1st ed.; Springer: Dordrecht, 2006; ISBN 978-0-387-28145-2.
12. Xu, T.; Li, J.H.; Momen, R.; Huang, W.J.; Kirk, S.R.; Shigeta, Y.; Jenkins, S. Chirality-Helicity Equivalence in the S and R Stereoisomers: A Theoretical Insight. *J. Am. Chem. Soc.* **2019**, *141*, 5497–5503, doi:10.1021/jacs.9b00823.
13. Guo, H.; Morales-Bayuelo, A.; Xu, T.; Momen, R.; Wang, L.; Yang, P.; Kirk, S.R.; Jenkins, S. Distinguishing and Quantifying the Torquoselectivity in Competitive Ring-Opening Reactions Using the Stress Tensor and QTAIM. *J. Comput. Chem.* **2016**, *37*, 2722–2733, doi:10.1002/jcc.24499.
14. Yang, P.; Xu, T.; Momen, R.; Azizi, A.; Kirk, S.R.; Jenkins, S. Fatigue and Photochromism S1 Excited State Reactivity of Diarylethenes from QTAIM and the Stress Tensor. *Int. J. Quantum Chem.* **2018**, *118*, e25565, doi:10.1002/qua.25565.
15. Xu, T.; Wang, L.; Ping, Y.; Mourik, T. van; Früchtl, H.; Kirk, S.R.; Jenkins, S. Quinone-Based Switches for Candidate Building Blocks of Molecular Junctions with QTAIM and the Stress Tensor. *Int. J. Quantum Chem.* **2018**, *118*, e25676, doi:10.1002/qua.25676.
16. Xu, T.; Momen, R.; Azizi, A.; Mourik, T. van; Früchtl, H.; Kirk, S.R.; Jenkins, S. The Destabilization of Hydrogen Bonds in an External

E-Field for Improved Switch Performance. *J. Comput. Chem.* **2019**, *40*, 1881–1891, doi:10.1002/jcc.25843.
17. Tian, T.; Xu, T.; van Mourik, T.; Früchtl, H.; Kirk, S.R.; Jenkins, S. Next Generation QTAIM for the Design of Quinone-Based Switches. *Chem. Phys. Lett.* **2019**, *722*, 110–118, doi:10.1016/j.cplett.2019.03.013.
18. Yang, W.; Zurbenko, I. Kolmogorov–Zurbenko Filters. *Wiley Interdiscip. Rev. Comput. Stat.* **2010**, *2*, 340–351, doi:10.1002/wics.71.
19. Tian, T.; Xu, T.; Kirk, S.R.; Rongde, I.T.; Tan, Y.B.; Manzhos, S.; Shigeta, Y.; Jenkins, S. Intramolecular Mode Coupling of the Isotopomers of Water: A Non-Scalar Charge Density-Derived Perspective. *Phys. Chem. Chem. Phys.* **2020**, *22*, 2509–2520, doi:10.1039/C9CP05879F.
